# Diphtheria

**Published:** 1883-08

**Authors:** 


### DIPHTHERIA.

We give the following summary and conclusion from a large number of replies to the question of the *Therapeutical Gazette* on this exceedingly important subject.

1st. Diphtheria may be either local or constitutional in its origin.

2d. It may continue as a purely local or purely constitution disease, or the local disease may be followed by constitutional affection, or *vice versa*—the disease in the vast majority of instances manifesting itself in both the constitutional disturbance and the local affection.

3d. The comparative value of local and constitutional remedies is dependent upon the nature of the affection—in individual cases.

4th. Diphtheria is a contagious disease, but liable to attack a healthy mucus membrane or to find an entrance through it into the circulation.

5th. The contagium of diphtheria is not a micrococcus, nor is it visible under the most powerful microscope yet manufactured.

6th. The contagion of diphtheria is of a gaseous nature—the result of decomposing fecal and other organic matter—and can be neutralized only by a true disinfectant, and not by an antiseptic.

7th. The best local application is the tincture of the chloride of iron. It may be supplemented by other applications, according to the indications in local cases.

8th. In a typical case of sthenic diphtheria administer large (10 grs.) and frequently (hourly) repeated doses of calomel, until the characteristic stools are secured. Following this give large doses of the tincture of chloride of iron every two hours, and administer alcohol within the limits of intoxication. In asthenic cases the calomel should be omitted and the main reliance be placed in the iron and alcohol.

